# *Streptococcus pyogenes* can support or inhibit growth of *Haemophilus influenzae* by supplying or restricting extracellular NAD^+^

**DOI:** 10.1371/journal.pone.0270697

**Published:** 2022-09-28

**Authors:** Hyunju Lee, Rebecca J. Edgar, Ian J. Lichtenstein, Jorge J. Velarde, Natalia Korotkova, Michael R. Wessels

**Affiliations:** 1 Division of Infectious Diseases, Boston Children’s Hospital, Boston, Massachusetts, United States of America; 2 Department of Pediatrics, Harvard Medical School, Boston, Massachusetts, United States of America; 3 Department of Pediatrics, Seoul National University Bundang Hospital, Seoul National University College of Medicine, Seoul, Korea; 4 Department of Molecular and Cellular Biochemistry, University of Kentucky, Lexington, Kentucky, United States of America; 5 Department of Microbiology, Immunology and Molecular Genetics, University of Kentucky, Lexington, Kentucky, United States of America; Universidade de Lisboa Faculdade de Medicina, PORTUGAL

## Abstract

Nicotinamide adenine dinucleotide (NAD^**+**^) is an essential co-factor for cellular metabolism and serves as a substrate in enzymatic processes. NAD^**+**^ is produced by *de novo* synthesis or salvage pathways in nearly all bacterial species. *Haemophilus influenzae* lacks the capacity for *de novo* synthesis, so it is dependent on import of NAD^+^ from the external environment or salvage biosynthetic pathways for recycling of NAD^**+**^ precursors and breakdown products. However, the actual sources of NAD^**+**^ utilized by *H*. *influenzae* in the respiratory tract are not well defined. In this study, we found that a variety of bacteria, including species found in the upper airway of humans, released NAD^**+**^ that was readily detectable in extracellular culture fluid, and which supported growth of *H*. *influenzae in vitro*. By contrast, certain strains of *Streptococcus pyogenes* (group A streptococcus or GAS) inhibited growth of *H*. *influenzae in vitro* by secreting NAD^**+**^-glycohydrolase (NADase), which degraded extracellular NAD^**+**^. Conversely, GAS strains that lacked enzymatically active NADase released extracellular NAD^**+**^, which could support *H*. *influenzae* growth. Our results suggest that many bacterial species, including normal flora of the upper airway, release NAD^**+**^ into the environment. GAS is distinctive in its ability to both release and degrade NAD^**+**^. Thus, colonization of the airway with *H*. *influenzae* may be promoted or restricted by co-colonization with GAS in a strain-specific manner that depends, respectively, on release of NAD^**+**^ or secretion of active NADase. We suggest that, in addition to its role as a cytotoxin for host cells, NADase may serve a separate function by restricting growth of *H*. *influenzae* in the human respiratory tract.

## Introduction

Nicotinamide adenine dinucleotide (NAD^**+**^) is an essential co-factor for both eukaryotic and prokaryotic organisms. NAD^**+**^ is a redox carrier that plays a critical role in cellular metabolism and energy-producing reactions associated with glycolysis, oxidative phosphorylation, and fermentation, and it serves as a substrate in enzymatic processes such as those catalyzed by ADP-ribosyl transferases and DNA ligases [1–3]. In bacteria, NAD^**+**^ homeostasis is maintained through *de novo* biosynthetic pathways that utilize amino acid substrates or salvage biosynthetic pathways for recycling of NAD^**+**^ precursors and breakdown products such as niacin, nicotinamide, and nicotinamide riboside [4,5].

Although the salvage pathway is not required for the growth of many bacterial pathogens, it is essential in several members of the *Haemophilus* genus including the important human pathogen, *Haemophilus influenzae*. *H*. *influenzae* is a fastidious, Gram-negative cocco-bacillus that inhabits the upper respiratory tract of human beings and has an obligate requirement for NAD^**+**^ (factor V) as well as heme (factor X) for growth [6–8]. The absolute requirement of *H*. *influenzae* for NAD^**+**^ is due to the lack of genes encoding the enzymes necessary for biosynthesis of NAD^**+**^ [9]. This observation raises the question, what is the source of NAD^**+**^ that permits growth of *H*. *influenzae* in its biological hosts? Under normal physiological conditions, low concentrations of NAD^**+**^ are found in plasma or lavage fluid from tracheal, laryngeal, and bronchial sources [10–12]. A study in a mouse model showed that NAD^**+**^ may be released into the extracellular space during tissue cytolysis by infectious or inflammatory processes or during membrane stress [13]. During invasive infection, *H*. *influenzae* might have the opportunity to use the nicotinamide adenine dinucleotide phosphate (NADP) pool of erythrocytes during bacterial multiplication in the blood stream [14]. Catalase, which contains both heme and NADPH and is widely distributed in mammalian tissues, is also proposed as a potential source of essential growth factors for *H*. *influenzae* [15]. However, the actual sources of NAD^**+**^ utilized by *H*. *influenzae* in the mammalian respiratory tract are not well defined.

We speculated that other members of the airway microbiota might serve as a source of exogenous NAD^**+**^ to support growth of *H*. *influenzae in vivo*. The plausibility of this hypothesis is supported by a study in chickens that reported infection of the upper airway by *Avibacterium paragallinarum* (previously *Haemophilus paragallinarum*) was dependent on co-colonization with *Staphylococcus chromogenes*, which released NAD^+^, a required factor for *A*. *paragallinarum* growth [16]. In the present study, we found that a variety of bacteria, including species resident in the upper airway of humans, released NAD^**+**^ that was readily detectable in extracellular culture fluid and which supported growth of *H*. *influenzae in vitro*. By contrast, the globally dominant invasive strain of the human pathobiont *Streptococcus pyogenes* (group A streptococcus or GAS) inhibited growth of *H*. *influenzae in vitro* by secreting NAD^**+**^-glycohydrolase (NADase), which degraded extracellular NAD^**+**^. Conversely, GAS strains that lacked enzymatically active NADase released extracellular NAD^**+**^, which could support *H*. *influenzae* growth. Our results suggest that many bacterial species, including normal flora of the upper airway, release NAD^**+**^ into the environment. We propose that GAS is distinctive in its ability to both release and degrade NAD^**+**^: colonization of the airway with *H*. *influenzae* may be promoted or restricted by co-colonization with GAS in a strain-specific manner that depends, respectively, on release of NAD^**+**^ or secretion of active NADase.

## Results

### Production of extracellular NAD^+^ by bacteria

**Fig 1:**
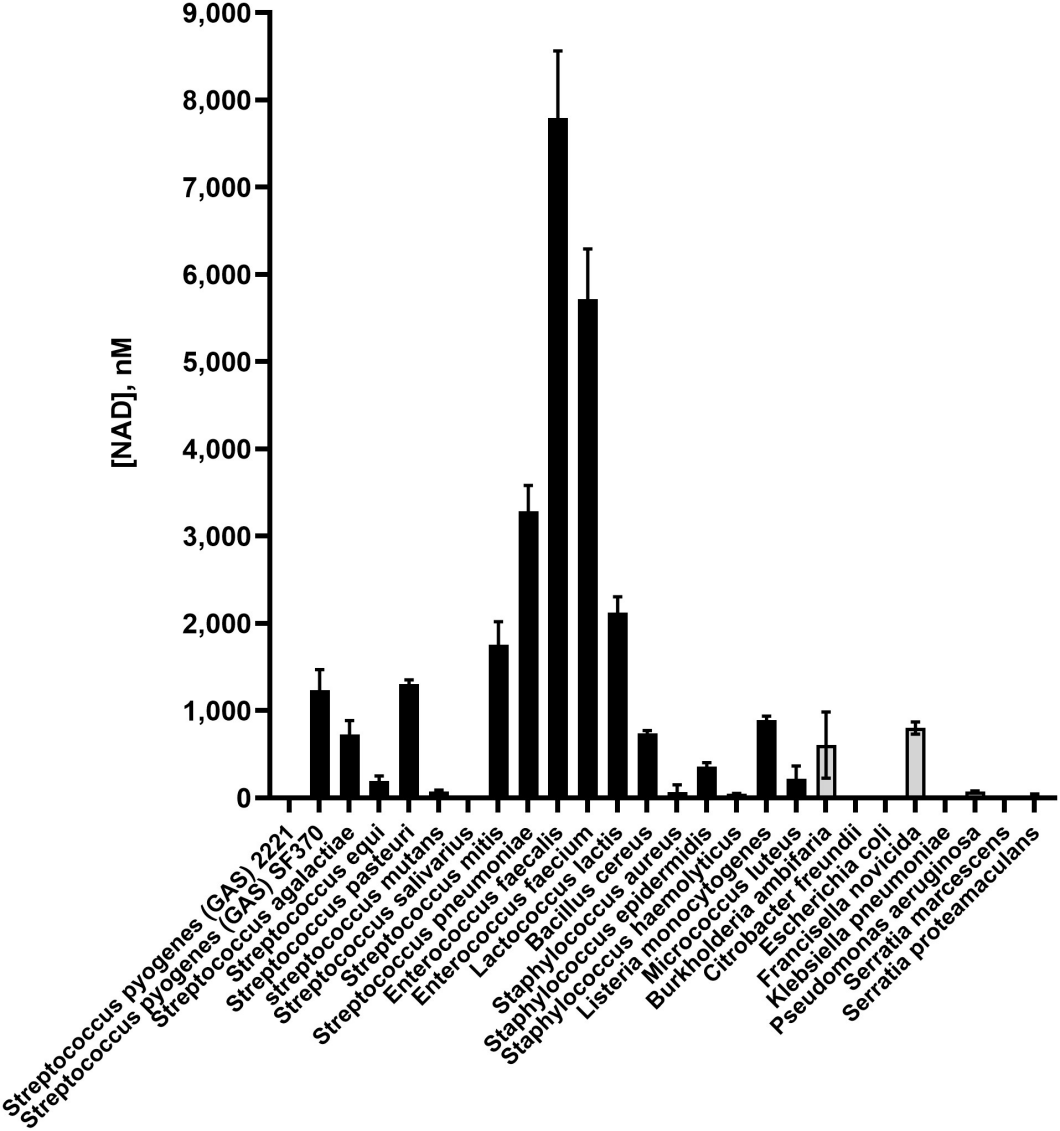
NAD^+^ is released into the growth medium by a variety of bacterial species. NAD^**+**^ concentration was measured in culture supernatants following bacterial growth for 16 hr. Gram-positive species are indicated by black bars and Gram-negative species by grey bars. Data are the mean of three biological replicates ± standard deviation.

To investigate the hypothesis that extracellular NAD^**+**^ is released by bacterial cells, we measured extracellular NAD^**+**^ production by various bacteria during growth *in vitro*. NAD^**+**^ was detected in culture fluid supernatants using the NAD/NADH-Glo^™^ assay. Among the bacterial species we tested, release of extracellular NAD^**+**^ was more common and usually in larger amounts among Gram-positive species, particularly enterococci and streptococci (Fig 1). The highest concentrations of extracellular NAD^**+**^ were found in cultures of *Enterococcus faecalis* and *E*. *faecium*, followed by some streptococcal and lactococcal species.

### GAS can both release and degrade extracellular NAD^+^

**Fig 2:**
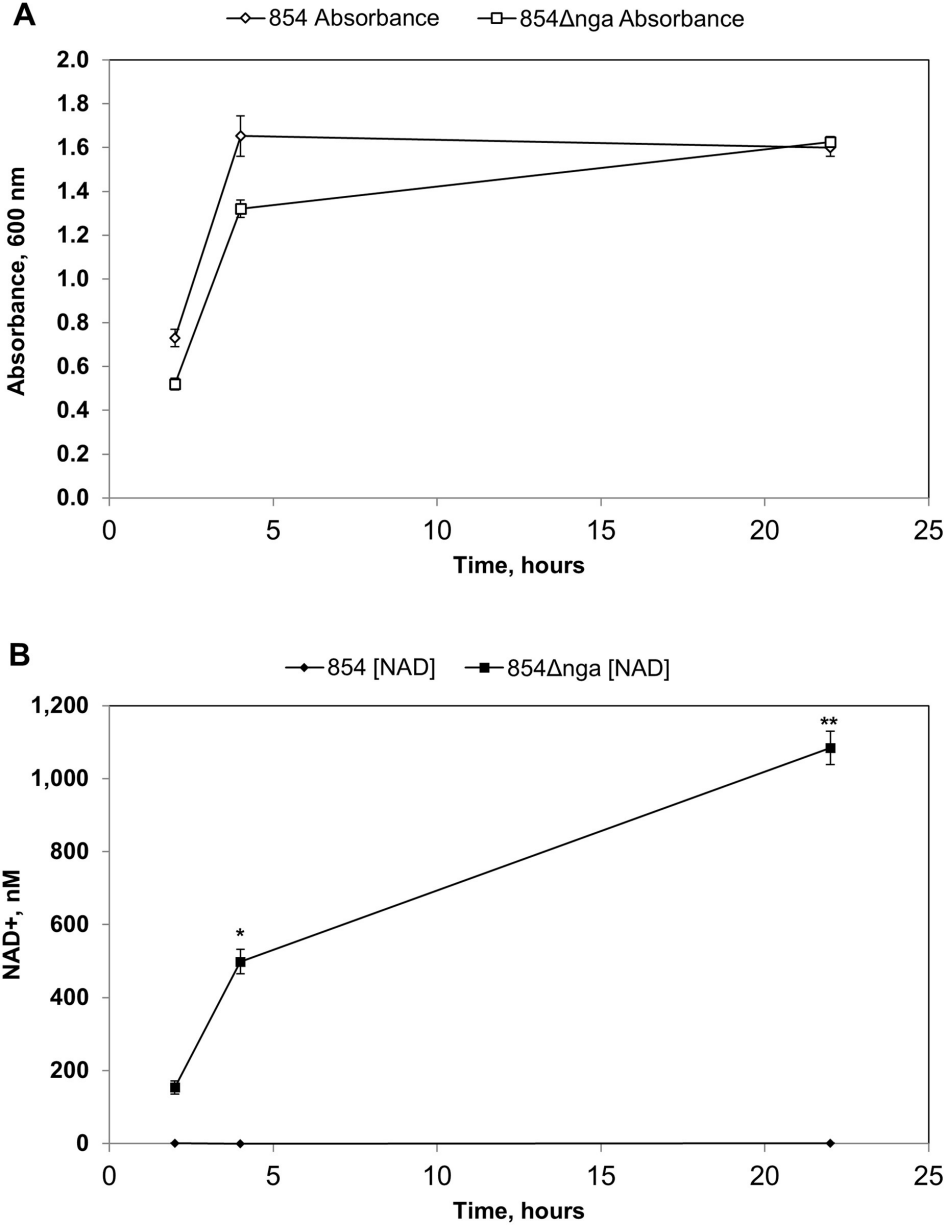
NAD^+^ is released into the growth medium by NADase-negative GAS strains. **A**. Growth curve of wild type GAS 854 and NADase-deficient 854Δ*nga*. **B**. Measurement of NAD^**+**^ concentration in the culture supernatant during growth of wild type GAS 854 and NADase-deficient 854Δ*nga*. Data are the mean of three biological replicates ± standard deviation. *P < 0.05; **P < 0.01.

The variable association of extracellular NAD^**+**^ with different strains of GAS was of particular interest since GAS has been shown to produce NAD^**+**^-glycohydrolase (NADase), a secreted enzyme that catalyzes the hydrolysis of NAD^**+**^ to nicotinamide and ADP-ribose [17]. NADase is thought to function as a co-toxin with the cholesterol-dependent cytolysin, streptolysin O (SLO). According to this model, GAS bacteria attach to host cells during infection. SLO secreted by adherent bacteria binds to the host cell membrane and mediates the translocation of NADase into the host cell cytoplasm [18,19]. Once inside the host cell, NADase degrades intracellular NAD^**+**^ and acts as a cytotoxin by depleting cellular energy stores [20,21]. We observed that extracellular NAD^**+**^ was undetectable in the culture supernatant of GAS strain 2221, which produces active NADase, but NAD^**+**^ was readily detected in cultures of strain SF370, which lacks NADase activity (Fig 1). We investigated this apparently reciprocal relationship between the presence of extracellular NAD^**+**^ and secretion of active NADase by measuring NAD^+^ in culture supernatants of a variety of GAS isolates that do or do not produce enzymatically active NADase (Table 1). NAD^+^ was detected in culture fluid of strains lacking active NADase but not in supernatants of strains that produced active NADase. We found also that extracellular NAD^+^ was not detected in cultures of GAS strain 854, a representative of the globally dominant M1T1 GAS clade that is a leading cause of GAS pharyngitis and invasive disease, nor in cultures of the M3 invasive clinical isolate 950771 (hereafter, 771). Both strain 854 and 771 produce active NADase. By contrast, NAD^+^ was abundant in cultures of their respective isogenic mutants, 854Δ*nga* and 771Δ*nga*, in which the gene for NADase (*nga*) is deleted (Table 1, Fig 2).

**Table 1:** NAD production and NADase activity of GAS strains. For NAD^+^ production, values represent mean±SD or the lower limit of detection for assays of three independent biological samples; for NADase activity, values represent mean endpoint titer (range) or the lower limit of detection for assays of three independent biological samples.

GAS strain	Description	NAD^+^ production (nMol/L)	NADase activity (arbitrary units)
SF370	Wild type M1	1234±238	<1
Manfredo	Wild type M5	849±207	<1
MGAS8232	Wild type M18	534±163	<1
Alab49	Wild type M53	1006±340	<1
87–282	Wild type M18	1897±227	<1
MGAS2221	Wild type M1	<10	19 (8,32)
MGAS10394	Wild type M6	<10	32 (32,32)
NZ131	Wild type M49	<10	13 (8,16)
854	Wild type M1	<10	21 (16,32)
854Δ*nga*	*nga* deletion mutant of 854	2529±359	<1
854*nga*G330D	*nga* point mutant of 854 with reduced NADase activity	136±54	<1
771	Wild type M3	<10	53 (32,64)
771Δ*nga*	*nga* deletion mutant of 771	1890±538	<1
771*nga*G330D	*nga* point mutant of 771 with reduced NADase activity	75±21	<1

**Fig 3:**
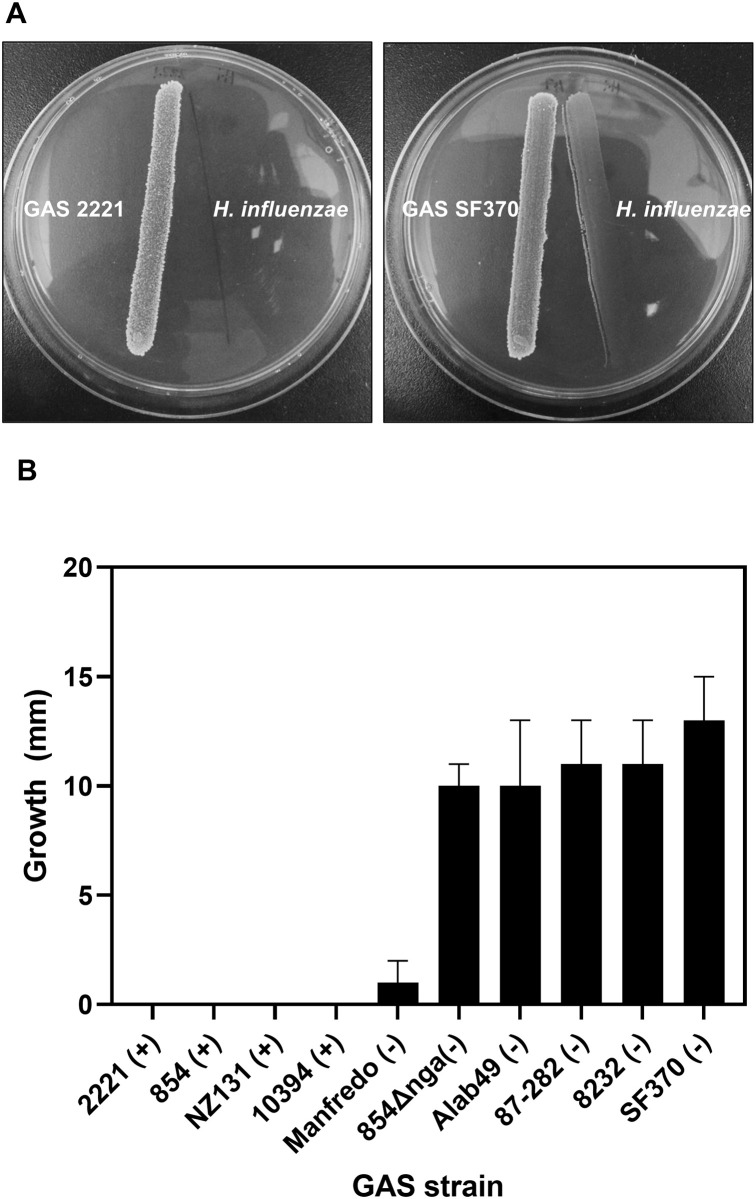
Certain GAS strains can support NAD^+^-dependent growth of *H*. *influenzae*. **A**. GAS 2221, NADase-positive, and GAS SF370, NADase-negative, were inoculated in a streak on hBHI agar (supplemented with heme but not NAD^**+**^**)** at a divergent angle from a streak of *H*. *influenzae* strain 2019. **B**. Growth of *H*. *influenzae* on NAD^**+**^-deficient BHI agar supported by disks inoculated with NADase-positive (+) or NADase-negative (-) GAS strains. Data represent width of the rings of *H*. *influenzae* growth surrounding the GAS disks and are the mean of three biological replicates ± standard deviation.

Since both GAS and *H*. *influenzae* are colonizers of the human upper airway, we wondered whether NADase-negative, but not NADase-positive, GAS could support growth of *H*. *influenzae* through release of extracellular NAD^**+**^. To test this idea in an *in vitro* system, we inoculated a streak of *H*. *influenzae* adjacent to a divergent streak of GAS on BHI agar supplemented with heme but not with NAD^**+**^. We found that a streak of NADase-negative GAS strain SF370 supported *H*. *influenzae* growth, whereas NADase-positive strain 2221 did not (Fig 3A). We also used a disk diffusion assay to test whether NAD^**+**^ released by GAS could support growth of *H*. *influenzae*. For these assays, *H*. *influenzae* was inoculated as a confluent lawn on BHI agar supplemented with heme but not NAD^**+**^, and then disks impregnated with bacterial suspensions of GAS were placed on the lawn. After overnight incubation, the area of *H*. *influenzae* growth around the disk reflected the release of NAD^**+**^ liberated into the medium by GAS (Fig 3B). NADase-negative GAS strains supported *H*. *influenzae* growth, whereas NADase-positive strains did not. Together, these results showed that NAD^**+**^ release by NADase-negative strains of GAS could serve as a source of NAD^**+**^ to support growth of *H*. *influenzae in vitro*.

### Inhibition of *H*. *influenzae* growth by NADase

**Fig 4:**
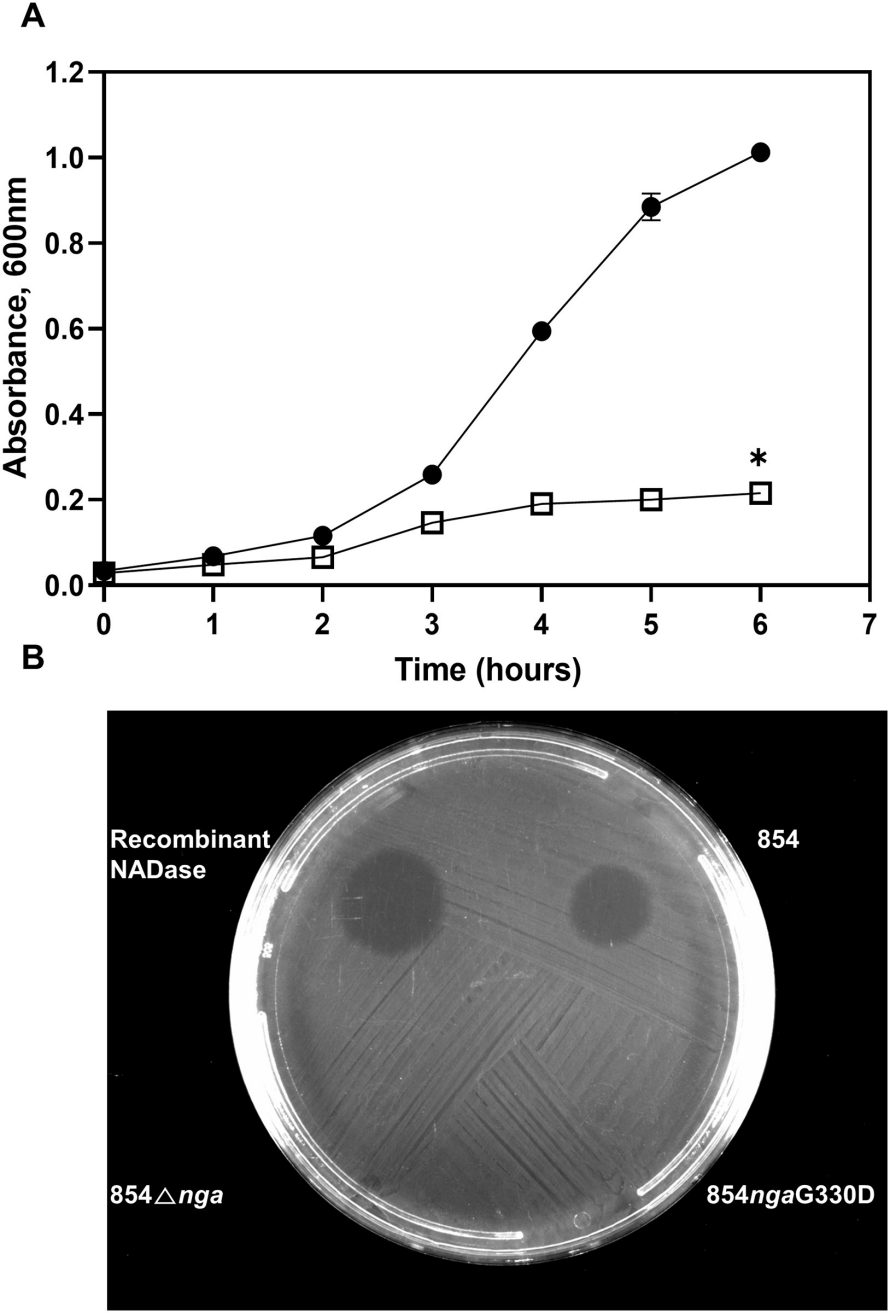
Effect of NADase on *H*. *influenzae*. **A**. Growth of *H*. *influenzae* in liquid medium in the absence (●) or presence (□) of recombinant NADase. Growth was inhibited in the presence of NADase (*P <0.05). Data are the mean±SD of three biological replicates (Error bars are <0.05 and are obscured by plot symbols for all data points except one at 5 hours). **B**. Bacterial lawn of *H*. *influenzae* shows inhibition of growth by recombinant NADase or GAS 854 culture supernatant.

We then examined whether NADase produced by GAS might inhibit growth of *H*. *influenzae* through depletion of extracellular NAD^**+**^. Initial experiments tested the effect of purified recombinant NADase in conditions favorable for growth of *H*. *influenzae*. Addition of recombinant NADase to liquid medium containing heme and NAD^+^ resulted in inhibition of growth of *H*. *influenzae* (Fig 4A).

The effect of NADase on *H*. *influenzae* growth was also tested by spotting aliquots of GAS culture supernatants on a lawn of *H*. *influenzae* grown on nutrient agar containing heme and NAD^**+**^. A zone of complete inhibition was seen with recombinant NADase or the supernatant of wild type 854. By contrast, no inhibition was seen in areas spotted with culture supernatants of the NADase deletion mutant 854Δ*nga* or 854*nga*G330D, which harbors a point mutation in the NADase active site that results in a reduction in enzymatic activity of >1000-fold [22] (Fig 4B). These results are further evidence that NADase produced by GAS inhibits growth of *H*. *influenzae* through NAD^**+**^ degradation.

### NADase-producing GAS inhibits, and NAD^+^-secreting GAS supports, *H*. *influenzae* growth in liquid culture

**Fig 5:**
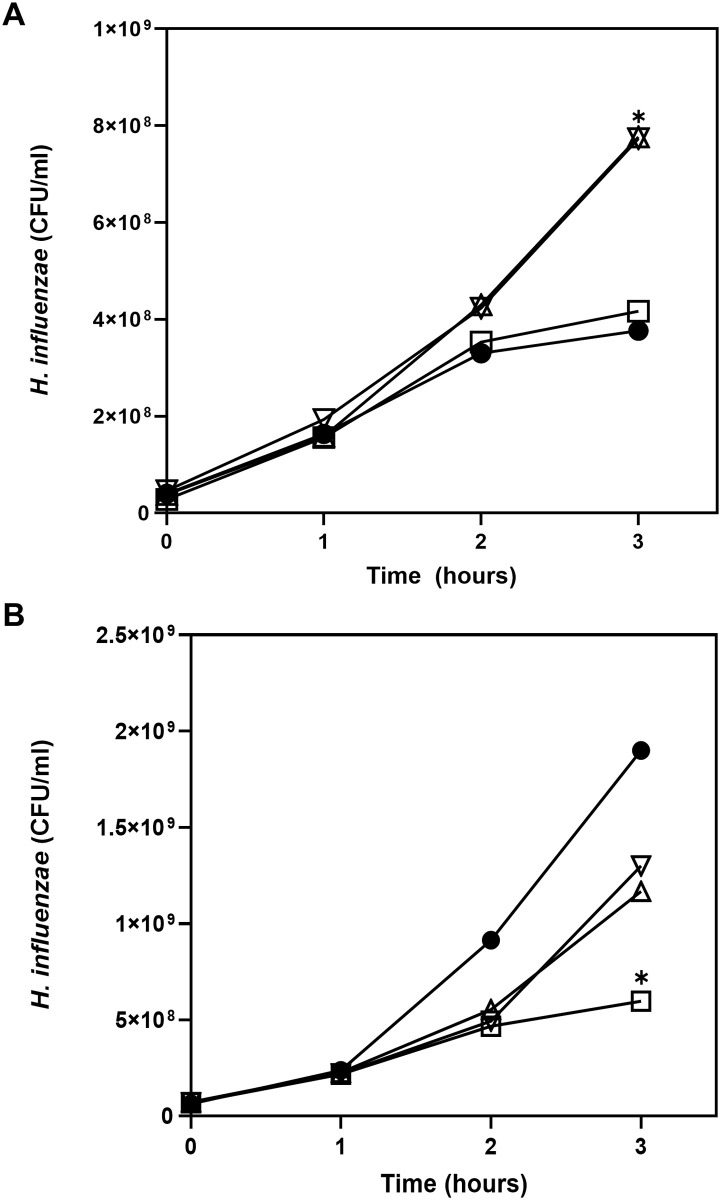
Effect of co-culture with GAS on growth of *H*. *influenzae*. **A**. Growth of *H*. *influenzae* alone (●) in BHI with heme (NAD^**+**^-depleted condition), or with GAS wild type strain 854 (□), 854*nga*G330D (Δ), or 854Δ*nga* (▽). *P <0.05, *H*. *influenzae* alone or with GAS 854 vs. *H*. *influenzae* with 854*nga*G330D or 854Δ*nga*. ***B***. Growth of *H*. *influenzae* alone (●) in sBHI (NAD^+^-replete condition) or with GAS 854 (□) or isogenic mutants 854*nga*G330D (Δ) or 854Δ*nga* (▽). *P <0.05, *H*. *influenzae* with 854 vs. *H*. *influenzae* alone, and *H*. *influenzae* with 854 vs. *H*. *influenzae* with 854*nga*G330D or 854Δ*nga*. Data are the mean of three biological replicates.

To further investigate the effects of GAS in supporting or inhibiting growth of *H*. *influenzae*, we inoculated the two bacterial species together in liquid cultures. In NAD^**+**^-deficient medium, *H*. *influenzae* exhibited increased growth when co-cultured with the NAD^**+**^-producing strains 854*nga*G330D or 854Δ*nga* compared to *H*. *influenzae* monoculture or growth with NADase-producing wild type GAS 854 (Fig 5A). By contrast, in NAD^**+**^-replete medium, *H*. *influenzae* growth was inhibited by co-culture with NADase-producing wild type GAS 854 compared to *H*. *influenzae* monoculture (Fig 5B). Under NAD^+^-replete conditions, co-culture with the NAD^**+**^-producing strains 854*nga*G330D or 854Δ*nga* also resulted in a reduction in *H*. *influenzae* growth, presumably as a result of nutrient competition, but this effect was significantly less than the inhibition produced by the NADase-producing wild type GAS 854. These findings support the hypothesis that bacteria, in this case, NADase-negative GAS strains, can serve as a source of extracellular NAD^**+**^, which is utilized by *H*. *influenzae*. Conversely, through depletion of NAD^**+**^, NADase produced by GAS inhibited growth of *H*. *influenzae* through degradation of extracellular NAD^**+**^.

## Discussion

NAD^**+**^ plays essential roles in cellular metabolism as a cofactor for redox reactions and as a consumable substrate of NAD^**+**^-dependent enzymes such as DNA ligases, protein deacetylases, and a variety of ADP-ribosyltransferases [4,23]. NAD^**+**^ is produced in bacteria by *de novo* synthesis or by recycling of NAD^**+**^ degradation products through synthetic pathways. However, some species such as *H*. *influenzae* lack the genes necessary for NAD^**+**^ biosynthesis and, therefore, are dependent on extrinsic sources. In this study, we examined the possibility that members of the host microbiota might serve as a source of NAD^**+**^ for *H*. *influenzae*. We found that extracellular NAD^**+**^ was released by a variety of bacteria commonly associated with the human microbiome, particularly by Gram-positive species. In our non-comprehensive survey, we found the highest amount of NAD^**+**^ release from *E*. *faecalis* and *E*. *faecium*, followed by *S*. *pneumonia*e and some streptoccoccal and lactococcal species.

It is not obvious how or why bacteria release NAD^**+**^ into the extracellular space. Possible mechanisms include active secretion, shedding in membrane vesicles, or passive diffusion through damaged cell membrane or from dead cells. Active release of NAD^**+**^ from viable bacteria implies that the process benefits the bacteria in some way, e.g., by cross-feeding other members of the same or another reciprocally beneficial species. Alternatively, or in addition, release of NAD^**+**^ might serve an adaptive function by feeding or signaling host cells to modify the producing organism’s ecological niche.

GAS is distinctive among the bacteria we tested in that we observed a reciprocal relationship between production of enzymatically active NADase and detection of extracellular NAD^**+**^ in GAS culture supernatants. This relationship implies that GAS releases NAD^**+**^ into the extracellular space and also can degrade extracellular NAD^**+**^ through secretion of NADase. The role of NADase in GAS pathogenesis has been investigated in detail. *In vitro* studies have provided evidence that NADase is translocated into host cells in a process that depends on SLO, and that NADase acts intracellularly to deplete host cell energy stores [18–20]. Emergence of a globally dominant invasive GAS M-type 1 clone in the 1980s was temporally associated with acquisition of DNA sequences encoding enzymatically active NADase and a highly active promoter driving expression of the NADase-SLO operon [24,25]. These genetic changes have been directly linked to enhanced virulence in experimental infection models [26].

A strength of our study is the identification of a potentially unique dual role of GAS in supporting or restricting growth of *H*. *influenzae* through the availability of NAD^+^. Additional strengths are quantitative assessments of NAD^+^ and NADase production by a diverse array of clinical isolates and verification of the results through use of two independent sets of isogenic GAS strains that produce or lack NADase.

A limitation of our study is that all experiments were performed *in vitro*. We did not assess the impact of host- and microbiota-related factors which may affect the interactions between GAS and *H*. *influenzae*. The host may play a significant role in providing NAD^+^ when hemolytic bacteria are present in the resident microbiota. The respiratory tract harbors a complex microbial community in which co-colonizing species may be in competing or symbiotic relationships [27]. *In vitro* studies found that production of hydrogen peroxide by *Streptococcus pneumoniae* inhibited the growth of *H*. *influenzae* [28]. Conversely, in a mouse model of co-infection, *H*. *influenzae* induced complement-mediated opsonophagocytic killing of *S*. *pneumoniae*, highlighting the complex nature of interspecies competition [29]. Further insight into the impact of NAD^+^- or NADase-producing GAS on *H*. *influenzae* awaits additional investigation, ideally under *in vivo* conditions that incorporate influences contributed by the host and microbiota.

Notwithstanding these limitations, the results of the current study suggest that, in addition to its role as a cytotoxin for host cells, NADase may serve a separate function by restricting growth of *H*. *influenzae* and possibly other competing flora in the human respiratory tract. Using several *in vitro* assays, we found evidence that NADase-negative strains of GAS could support growth of *H*. *influenzae* and that NADase-positive GAS could inhibit *H*. *influenzae* growth. It seems likely that similar interspecies dynamics could occur in the human upper airway. Our findings that many human-associated bacteria release NAD^**+**^, and that GAS may support or restrict NAD^**+**^-dependent growth of *H*. *influenzae*, open a promising avenue for further investigation of interspecies cooperation and competition in the human-associated microbiota.

## Methods

### Bacterial strains and growth conditions

Bacterial strains used in this study are shown in Table 2. GAS 854 is an M type 1 strain isolated from a patient with a retroperitoneal abscess [30]. GAS 950771 (abbreviated here as 771) is an M type 3 strain isolated from a child with necrotizing fasciitis and sepsis [31].

**Table 2:** Bacterial strains used in this study.

Strain	Description	Reference/source
*Haemophilus influenzae*		
Eagan-Sm	Type b, streptomycin-resistant	[29]
2019	Non-typeable clinical isolate (chronic obstructive pulmonary disease)	[32]
*Streptococcus pyogenes*		
854	Serotype M1T1, clinical isolate (retroperitoneal abscess)	[30]
854*nga*G330D	Asp substitution at Gly330 of NADase; catalytic activity reduced >1000-fold	[33]
854Δ*nga*	*nga* deletion mutant	[33]
950771 (771)	Serotype M3, clinical isolate (necrotizing fasciitis)	[31]
771Δ*nga*	*nga* deletion mutant	[18]
771*nga*G330D	Asp substitution at Gly330 of NADase; catalytic activity reduced >1000-fold	This study
MGAS 2221	Serotype M1T1, clinical isolate (pharyngitis)	[25]
SF370	Serotype M1T1, clinical isolate (wound infection)	[34]
MGAS8232	Serotype M18, clinical isolate (acute rheumatic fever)	[35]
87–282	Serotype M18, clinical isolate (acute rheumatic fever)	[36]
Manfredo	Serotype M5, clinical isolate (acute rheumatic fever)	[37]
MGAS10394	Serotype M6, clinical isolate (pharyngitis)	[38]
NZ131	Serotype M49, clinical isolate (glomerulonephritis)	[39]
Alab49	Serotype M53, clinical isolate (impetigo)	[40]
*Escherichia coli*	DH5α	Zymo Research
*Streptococcus agalactiae*	D013-03	Dr. E. Sokurenko
*Streptococcus equi*	CF32	[41]
*Streptococcus mitis*	NS51	ATCC
*Streptococcus mutans*	UA159	ATCC
*Streptococcus pasteuri*	D021-19.2	Dr. E. Sokurenko
*Streptococcus pneumoniae*	TIGR4	[42]
*Streptococcus salivarius*	d063-46.2	Dr. E. Sokurenko
*Bacillus cereus*	ATCC 14579	ATCC
*Enterococcus faecalis*	d018-28	Dr. E. Sokurenko
*Enterococcus faecium*	d011-49	Dr. E. Sokurenko
*Lactococcus lactis ssp lactis*	C2	[43]
*Listeria monocytogenes*	EGDe	Dr. C. Gahan
*Micrococcus luteus*	ATCC 4698	ATCC
*Staphylococcus aureus*	d030-04	Dr. E. Sokurenko
*Staphylococcus epidermidis*	ATCC 12228	ATCC
*Staphylococcus haemolyticus*	d003-11	Dr. E. Sokurenko
*Burkholderia ambifaria*	MC40-6	US DOE Joint Genome Institute
*Citrobacter freundii*	d007-08.2	Dr. E. Sokurenko
*Francisella novicida*	GA99-3548	Dr. S.K. Urich
*Klebsiella pneumoniae*	d002-04-1	Dr. E. Sokurenko
*Pseudomonas aeruginosa*	d023-16.1	Dr. E. Sokurenko
*Serratia marcescens*	d019-36.1	Dr. E. Sokurenko
*Serratia proteamaculans*	568	Dr. D. van der Lelie

GAS was grown in Todd-Hewitt broth (BD Difco) supplemented with 0.5% yeast extract (THY) or on THY agar supplemented with 5% defibrinated sheep blood (Fisher Scientific). In some assays GAS was grown in brain heart infusion (BHI) broth (BD Difco) supplemented with 10 μg mL^-1^ heme (hBHI). A stock solution of hemin was made by dissolving 50 mg of bovine hemin (Sigma-Aldrich #H9039) in 5 ml of 0.1 N NaOH. Five ml of hemin solution was mixed vigorously with 5 ml of ethylene glycol and added to 10 ml of 5X Tris-Borate buffer and 30 ml of water. The final solution was filter-sterilized and stored at 4ºC in a foil-wrapped flask for up to 1 month.

The *H*. *influenzae* strains used in these studies are a streptomycin-resistant mutant of *H*. *influenzae* type b strain Eagan provided by Jeffrey Weiser and non-typeable strain 2019 provided by Margaret Ketterer [29,32]. *H*. *influenzae* was grown in BHI broth supplemented with 2% Fildes enrichment (Fisher Scientific, sBHI) unless otherwise specified. *Escherichia coli* DH5α was used as a host for molecular cloning (Zymo Research) and was grown in Lysogeny Broth (LB) medium (Novagen). When appropriate, antibiotics were used at the following concentrations: streptomycin 50 μg mL^-1^, gentamicin 5 μg mL^-1^, erythromycin 200 μg mL^-1^ or 1 μg mL^-1^ for *E*. *coli* or GAS, respectively.

### DNA manipulations

To generate a GAS mutant that produces NADase harboring a G330D substitution, GAS strain 771 was transformed by electroporation with plasmid pJRS233-*nga*G330D (25). The resulting transformants then underwent allelic exchange to replace the wild type *nga* gene with the mutated allele as previously described [44]. Measurement of NADase activity in culture supernatants confirmed that the 771*nga*G330D mutant expresses NADase with enzymatic activity below the limit of detection in our assay [18].

### Measurement of extracellular NAD^+^

To determine the concentration of extracellular NAD^+^, all bacterial strains except *Micrococcus luteus* were grown at 37°C. Gram-positive and Gram-negative bacteria were grown in THY medium without aeration and LB medium with aeration, respectively. *Micrococcus luteus* was grown in LB medium with aeration at 30°C. The supernatant was collected by centrifugation (16,000 g, 10 min) from the overnight-grown bacteria and filtered through a 0.22-μm-pore-size filter to remove residual bacterial cells. The concentration of NAD^+^ in the supernatant samples was determined by NAD/NADH-Glo^™^ assay (Promega) according to the manufacturer’s protocol. The samples were either processed immediately or stored at -80°C before the assay. The first step of the protocol is the addition of 0.4 N HCl and heating at 60°C for 15 min, which inactivates NADase enzymatic activity.

### Measurement of NADase activity

NADase activity in GAS culture fluid supernatants was assayed as described previously [18,45,46]. Enzyme activity was expressed as the reciprocal of the fluid dilution that depleted 50% of the NAD^+^ substrate.

### Disk diffusion assay for effect of bacterial NAD^+^ release on growth of *H*. *influenzae*

*H*. *influenzae* strain 2019 was grown in BHI broth supplemented with 10 μg mL^-1^ heme and 10 μg mL^-1^ β-NAD^+^ (Sigma-Aldich, N7381; heme- and NAD^+^-supplemented BHI; hnBHI). To test *H*. *influenzae* growth in the disk diffusion assay, the overnight-grown culture was diluted to A_600nm_ 0.3 and spread with a sterile cotton swab across a BHI broth supplemented with 10 μg mL^-1^ heme (hBHI) agar plate. GAS strains were grown overnight in BHI medium, diluted to (A_600nm_ 0.9–1), and 10 μl aliquots of the bacterial suspension were dispersed onto 6 mm paper disks (BD, 231039). Disks were placed on the hBHI agar plate prepared as described above. The negative control was an empty disk. The positive controls were prepared by pipetting 10 μl of 0.3 or 0.6 nM β-NAD^+^ onto the disks. The plates were incubated overnight at 37°C in room air supplemented with 5% CO_2_.

### Recombinant NADase

Recombinant GAS NADase was produced in *E*. *coli* and purified as described previously except that Q Sepharose chromatography was omitted [46].

### Liquid culture growth inhibition assay

To determine the effect of NADase on the growth of *H*. *influenzae* in liquid culture, *H*. *influenzae* strain Eagan-Sm was grown in sBHI or sBHI supplemented with 2 μM purified recombinant NADase.

### Agar plate growth inhibition assay

A bacterial lawn was prepared by evenly spreading a suspension of *H*. *influenzae* strain Eagan-Sm from a mid-exponential phase culture (A_600nm_ 0.3–0.4) on a sBHI agar plate using a sterile cotton swab. Supernatants were obtained from GAS broth cultures at late-exponential phase (A_600nm_ 0.8–0.9). Cells were removed from the culture fluid by centrifugation, and the supernatant was sterile-filtered and concentrated 30-fold in a Vivaspin 20 spin concentrator with a 10-kDa-molecular-mass cutoff (GE Healthcare). Five μL aliquots of concentrated supernatant or of 2 μM purified recombinant NADase were applied to the plates, which were then incubated at 37°C for 16 hr.

### Co-culture assays

Co-culture assays were performed as previously described with minor modifications [28]. Briefly, GAS strains were grown in THY broth at 37°C to mid-exponential phase (A_600nm_ 0.3–0.4), collected by centrifugation, and then resuspended in the same volume of fresh THY. *H*. *influenzae* strain Eagan-Sm was grown in sBHI at 37°C to mid-exponential phase (A_600nm_ 0.3–0.4), collected by centrifugation, washed in PBS, and then resuspended in fresh sBHI for assays to study the effect of NADase or in hBHI to study the effect of NAD^+^. Equal volumes, containing approximately 10^7^ CFU of *H*. *influenzae* and 10^8^ CFU of GAS were then mixed and incubated at 37°C in 96-well polystyrene microtiter plates. As a negative control, *H*. *influenzae* was mixed with an equal amount of THY broth without GAS. After various periods of incubation, replicate aliquots were removed and serially diluted in PBS for quantitative culture on sBHI-streptomycin selective agar plates for *H*. *influenzae* or blood agar plates for GAS.

### Statistical analysis

Statistical significance of differences between experimental groups was determined by unpaired student’s t-test. *P* values of less than 0.05 were considered significant and are indicated by an asterisk (*) in figures.

## Supporting information

S1 Data Fig 1 primary data. (XLSX)

S2 Data Fig 2 primary data. (XLSX)

S3 Data Fig 3b primary data. (XLSX)

S4 Data Fig 4A primary data. (XLSX)

S5 Data (ZIP)
